# Large-scale comparative neuroimaging: Where are we and what do we need?

**DOI:** 10.1016/j.cortex.2018.11.028

**Published:** 2019-09

**Authors:** Michel Thiebaut de Schotten, Paula L. Croxson, Rogier B. Mars

**Affiliations:** a Brain Connectivity and Behaviour Group, Sorbonne Universities, Paris France; b Frontlab, Institut du Cerveau et de la Moelle épinière (ICM), UPMC UMRS 1127, Inserm U 1127, CNRS UMR, Paris, France; c Groupe d’Imagerie Neurofonctionnelle, Institut des Maladies Neurodégénératives-UMR 5293, CNRS, CEA University of Bordeaux, Bordeaux, France; d Friedman Brain Institute, Icahn School of Medicine at Mount Sinai, New York, NY, USA; e Mortimer B. Zuckerman Mind Brain Behavior Institute, Columbia University, New York, NY, USA; f Wellcome Centre for Integrative Neuroimaging, Centre for Functional MRI of the Brain (FMRIB), Nuffield Department of Clinical Neurosciences, John Radcliffe Hospital, University of Oxford, Oxford, United Kingdom; g Donders Institute for Brain, Cognition and Behaviour, Radboud University Nijmegen, Nijmegen, Netherlands

**Keywords:** Comparative neuroscience, Neuroimaging, Primate, Macaque, Connectivity

## Abstract

Neuroimaging has a lot to offer comparative neuroscience. Although invasive “gold standard” techniques have a better spatial resolution, neuroimaging allows fast, whole-brain, repeatable, and multi-modal measurements of structure and function in living animals and post-mortem tissue. In the past years, comparative neuroimaging has increased in popularity. However, we argue that its most significant potential lies in its ability to collect large-scale datasets of many species to investigate principles of variability in brain organisation across whole orders of species—an ambition that is presently unfulfilled but achievable. We briefly review the current state of the field and explore what the current obstacles to such an approach are. We propose some calls to action.

Neuroimaging using magnetic resonance imaging (MRI) has been around for a few decades and has established itself as one of the primary tools for understanding brain structure and function. More recently, it has been used as a tool not just to understand the human brain, but also to compare the structure and function of different species’ brains. Some studies in this special issue of Cortex illustrate these developments, using neuroimaging to compare connections (Eichert et al., 2018), sulcal patterns (Margiotoudi et al., 2019), cortical thickness (Hopkins, Latzman, Mahovetz, Li, & Roberts, 2019), and volumes to surface ratios (Heuer et al., 2019). These papers demonstrate how new developments in MRI are readily adopted in comparative studies and how they have been instrumental in its development. Presently, however, this is still a niche endeavour. In this communication, we explore where the field of comparative neuroimaging is, what steps need to be taken to scale it up, and why we would want to do so.

One factor that has driven progress in the field of comparative neuroscience is that we are approaching a consensus regarding the value that MRI can add to our understanding of anatomy. While it does not exactly reflect the measures that traditional techniques such as cytoarchitectonics (Herold et al., 2019, Palomero-Gallaghera and Zilles, 2019) and tract tracing (Borra & Luppino, 2019) do, it does reflect a great deal more than just noise. With the right experimental design and the right sample size, we can take advantage of MRI to advance our understanding of the brain in a comparative and evolutionary perspective. MRI provides us with the ability to assess multiple modalities, such as structural and functional domains, and multiple species, including rare species and humans that may not otherwise be accessible with traditional methods. Comparative MRI also shows a great deal of promise as a tool that can be combined with other comparative neuroscience datasets, such as behaviour, cell composition, neural function, and genetics.

To achieve novel, robust, reproducible findings, comparative MRI must move beyond small-scale case studies. For some time now, the field of comparative MRI has been a small and undervalued one, comprised of a few researchers with a high degree of technical expertise and the time and resources to gather high quality data. By contrast, the field of human MRI has vast quantities of researchers and resources and massive datasets acquired with high quality tools. While comparative MRI will never be as large or busy a field, we argue that it can make more significant and meaningful contributions to our understanding of the brain by exploiting its strengths and move towards scaling up, which will most likely be achieved through collaboration and teamwork.

Here we examine some practicalities of generating and using such large datasets. We focus mainly on forming a set of standards that we can use as a field. These standards include the sharing of data and the development of standardised tools. We also call for agreement on a common framework for understanding how to compare across diverse species and for agreeing on approaches that will allow us to ask genuinely novel and exciting questions about the guiding principles of the brain. We will focus mainly on primate comparative neuroscience but acknowledge that rapid advances are made in studying other mammals, including rodents (e.g. Berns et al., 2015, Grandjean et al., 2017).

## 1 Part I: why comparative MRI?

Before discussing the current state of comparative neuroimaging, it is worthwhile to take a step back and ask why one would want to invest in neuroimaging at all when one is interested in comparative neuroscience. Neuroimaging has been criticised for not having the same resolution or direct access to data as some of the "gold standard" methods and, similar to any novel method, has methodological issues that deserve attention (Maier-Hein et al., 2017, Reveley et al., 2015, Zilles and Amunts, 2015). However, there are many reasons why imaging should be considered seriously in comparative neuroscience.

### 1.1 Non-invasive, repeatable, and multi-modal probing

MRI is a powerful tool in that it allows us to acquire whole-brain data across multiple modalities, non-invasively and repeatedly. It relies on the properties of some atomic nuclei to absorb and emit radio frequency energy when placed in an external magnetic field. It allows one to collect data from the whole brain in a relatively short time, usually calculated in minutes or at most hours. By doing so, we can pursue hypotheses that go beyond a single brain region or circuit and can use data-driven, as well as hypothesis-driven approaches. The technique is non-invasive, so can be used without harm to the animal or without destroying tissue.

The non-destructive nature of imaging also makes it possible to collect data of multiple modalities. Different sequences are sensitive to different features of a sample's tissue, allowing the researcher to assess very different aspects of brain anatomy and function. For instance, the primary distinction is, of course, between structural measures and measures of brain activation. Measures sensitive to brain activation, such as the BOLD contrast (Ogawa, Lee, Kay, & Tank, 1990), can show how the brain responds to different stimuli or be probed in the absence of a task to determine the covariation of activity across regions (e.g. Vincent et al., 2007). Within the structural modality there are many different possibilities. Most popular are standard T1-weighted “grey matter” images. “White matter” imaging can be performed using diffusion MRI, looking at the microscopic displacement of water molecules, and following displacement to reconstruct the course of white matter fibres (Basser, Mattiello, & Le Bihan, 1994). Diffusion MRI is also now increasingly used to look at properties of the grey matter (Fukutomi et al., 2018). More recently, the sensitivity of MRI sequences to other tissue properties such as the presence of myelin and iron has been exploited (Glasser and Van Essen, 2011, Weiskopf et al., 2013). These examples demonstrate that, through repeated scanning using different sequences, neuroimaging allows the direct comparison of different tissue properties from the same brain.

The ability to scan repeatedly also allows one to examine the same brain multiple times using the same sequences, but after a manipulation of the brain or tissue. For instance, examining plasticity effects in the animal brain using comparative imaging would benefit our understanding of the human brain (Assaf, Johansen-Berg, & Thiebaut de Schotten, 2017). As an example of this approach, the recent identification by MRI of similar mechanisms of plasticity in mice and humans allowed further characterisation of these results with histology in mice and the extension of the conclusion to humans (Sagi et al., 2012). The same idea can be applied to study changes in brain activity between hemispheres following surgical lesions such as sectioning of the corpus callosum (O'Reilly et al., 2013) or lesions to the specific brain areas such as the hippocampus (Croxson et al., 2012, Croxson et al., 2011, Froudist-Walsh, Browning, Young, et al., 2018, Froudist-Walsh, Browning, Croxson, et al., 2018). It is also possible to study differences in brain organization as a result of placing it in different environments. For instance, recent studies sought to investigate the neuroimaging effects of social enrichment (Diamond, Krech, & Rosenzweig, 1964) on captive macaques housed in different-sized social groups. By combining different modalities, they could show both structural changes in grey matter content of specific brain areas and changes in the interactions between cortical areas, in particular cortical networks (Mars et al., 2012, Noonan et al., 2014, Sallet et al., 2011).

In sum, imaging allows repeated and multi-modal probing of the whole brain without harm to subjects or damage to tissue.

### 1.2 Digital, reusable, manipulable datasets

One of the greatest strengths of neuroimaging datasets is that they are digital, and therefore permanent, manipulable, reusable, and shareable. The permanency and reusability mean that the data can be used again and again for many purposes besides the one for which they were initially intended. For instance, data that are designated “control” datasets for experiments involving manipulations in non-human primates or rodents, or post-mortem data sets collected at the end of a study, can be used again for comparative work at no additional grant or animal costs. These data can be shared publicly, and it is potentially possible to combine multiple datasets even if they are acquired at different sites with different hardware and parameters. Since whether and how to do this is a complicated question, we discuss this in more detail in Part III.

The digital nature of the data means that one can analyse them within statistical frameworks that were previously impossible for non-human data. For instance, Croxson et al. (Croxson, Forkel, Cerliani, & Thiebaut de Schotten, 2017) recently demonstrated that the variability in brain organisation across several macaque monkeys and several humans is present in the regions that show the most expansion in the human compared to the macaque, arguing for a role in within-species flexibility to achieve between-species differentiation. Similarly, digital manipulation of cortical images allows one to directly compare relative expansion of parts of the cortex and to compare expansion during ontogeny with that during phylogeny (Hill et al., 2010). These approaches open the way for quantitative comparative neuroscience, which we will discuss further in Parts II and III.

### 1.3 Like-for-like comparisons across a large range of species

The most significant advantage of MRI is that it is one of the few techniques that can truly bridge the gap between species by providing the means to acquire data from multiple species using the same non-invasive method.

**Fig. 1:**
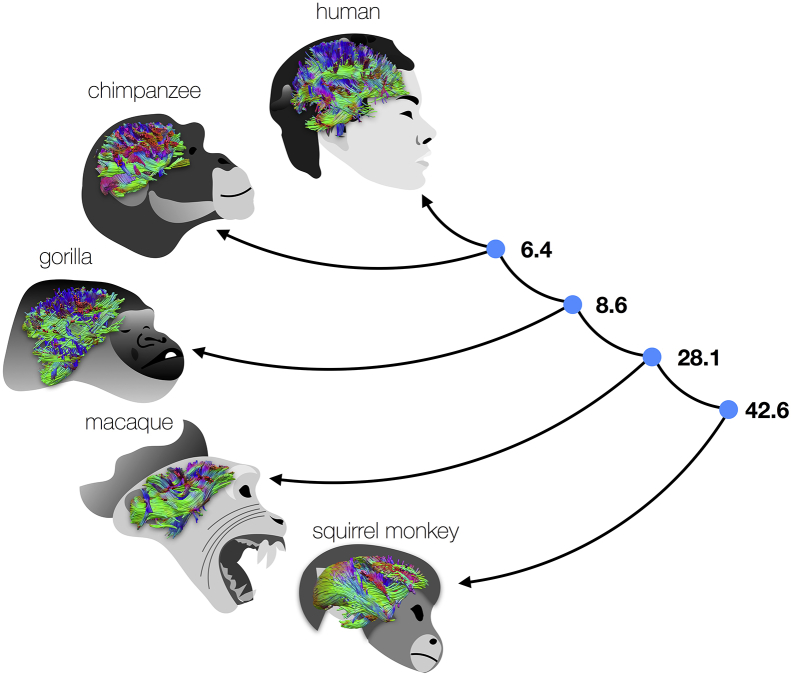
Comparing white matter connections across primates shed lights on brain evolution. Numbers on the right indicate millions of years that separate species from their common ancestor (blue dots). White matter connections are colour-coded in green for anteroposterior, red for medial-lateral and blue for ventral-dorsal.

The aims of comparative neuroscience studies fall broadly into two categories: to identify the guiding principles that govern brain structure and function across species, and to identify the things that are specific to a particular species and learn why. Since traditional techniques for studying neural anatomy are time consuming and invasive, most studies rely on comparisons between a limited number of species. However, if we want to understand the *principles* behind brain/behaviour relationships, we need to move towards studies with large enough datasets to find new results using exploratory and hypothesis-driven analysis techniques to generalise findings across members of various species' and to examine variability between individuals of the same species. The need to compare maps of brain organisation across species was recently eloquently expressed in a report by Striedter and colleagues (Striedter et al., 2014) and MRI has the potential to achieve this goal (Fig. 1).

Many comparisons across species to date, especially when involving the human brain, by necessity compare data obtained using different techniques. For example, comparisons of brain activity relied on single-unit recordings in monkeys and on functional MRI in humans, while comparisons of connections depended on tracer data in monkeys and blunt dissections in humans. The non-invasive and repeatable nature of MRI allows us to compare like with like. The multi-modal nature of imaging then allows one to obtain maps that reflect multiple aspects of brain organisation that can be compared or eventually combined.

As we will demonstrate below, cortical cartography of the human brain using neuroimaging is now well established, as demonstrated by a recent new multi-modal map of the human brain (Glasser et al., 2016), and comparisons with non-human primate maps obtained in a similar fashion are increasingly attempted. In the ideal case, this involves identical equipment, acquisition sequences, resolution, and analysis methods. In practice, we achieve parity in some measures, mainly from a theoretical perspective. By using a rigorous approach in which prior information, for example, from histological and tract tracing studies, is taken into account, we can draw sensible conclusions about the relationship between species.

## 2 Part II: where are we with comparative MRI?

In this section, we provide a summary review of some of the ways neuroimaging has been used in comparative neuroscience, without aiming to be exhaustive (see Mars et al., 2014, Rilling, 2014, for more comprehensive overviews).

Three reasons have motivated the use of neuroimaging to compare species in most of the recently published studies. These are (1) validating novel methods to examine the brain, (2) identifying qualitative similarities and differences across species, and (3) comparing brains of different species using quantitative measures.

### 2.1 Validation of novel methods to examine the brain

Invasive studies on living human brains are mostly considered unethical. The advent of the MRI allowed for a measure of specific features that were only available in animals before. However, this inevitably led to discussions of how the MRI-based measures obtained in the human compared to the data obtained using invasive techniques in experimental animals. Some of the primary uses of comparative neuroscience are aimed to address this issues, either by studying the human and the non-human animal using the same technique to confirm comparisons across techniques or to directly compare the invasive and MRI-based results in the same subject.

A prime example of the latter is the simultaneous intracortical recordings of neural signals and blood oxygen level dependent functional MRI in macaques first pioneered by Logothetis (Logothetis, Pauls, Augath, Trinath, & Oeltermann, 2001), but similar studies can be undertaken to validate structural markers.

Typical comparative neuroscience approaches mapping the type and distribution of neurons or neurotransmitter receptors across the brain is, at present, simply not possible using neuroimaging. However, some sequences are sensitive to the presence of specific tissue properties. For instance, many studies noted that the intensity of a T1 scan reflected the content of cortical myelin across areas (e.g. Bock et al., 2013, Geyer et al., 2011). Using different imaging protocols, various groups have quantified cortical myelin (Glasser and Van Essen, 2011, Lutti et al., 2014) and demonstrated its replicability across species, as well as its potential usefulness for comparative neuroscience (Glasser et al., 2013). Qualitative comparisons between retinotopic and tonotopic maps initially identified in macaques with histology and myelin-sensitive imaging in humans validated these approaches (Dick et al., 2012, Sereno et al., 2013). A more direct comparison comes from Large et al. (2016), who scanned macaques using a myelin-sensitive sequence to identify cortical area MT and then used traditional histology to validate their results.

The same logic was applied to brain connectivity analyses. Traditional brain connectivity studies require several steps, such as injection of tracers in vivo, sacrifice, brain slicing, time-consuming observations, and skilful but subjective drawings (Schmahmann & Pandya, 2006), most of which would be considered unacceptable in human samples. Tractography of diffusion-weighted MRI (dMRI) data by piecing together local estimates of water diffusion allowed for the depiction of gross white matter organisation for the first time in the living human brain (Catani & Thiebaut de Schotten, 2012). At first, the comparison between primate tracing studies and human tractography was employed as a method of validating qualitatively novel findings in human brain anatomy. For instance, Makris et al. (2005) used side-by-side comparisons of coronal slices of axonal tracing in monkeys and diffusion maps to delineate the probable location of the three branches of the superior longitudinal fasciculus in humans. Later, methodological advances in tractography allowed for the more detailed reconstruction of the course of these tracts in humans, identifying hemispheric differences (Thiebaut de Schotten et al., 2011). Similarly, the middle longitudinal fasciculus (Makris et al., 2009), the stratum proprium of the interparietal sulcus (Uesaki, Takemura, & Ashida, 2018), and the fronto-tectal tract (Quentin, Chanes, Migliaccio, Valabregue, & Valero-Cabre, 2013) were defined and reconstructed in humans by dint of their correspondence with histological tracing in primates.

Quantitative validation of fibre connectivity strength also came from the comparison of axonal tracing and the tractography-derived estimation of fibre strength (although strength of connections is difficult to quantify, e.g., Rockland, 2015). For example, preliminary findings at first reported a weak but significant correspondence between the number of tractography trajectories reconstructed and the actual strength of the connection derived from histology (i.e., about 9% of the shared variance between streamline density and CoCoMac structural connectivity tract strength) (van den Heuvel et al., 2015). This number was then further improved using mathematical adjustments such as correction for distance and logarithmic transformation of the number of trajectories reconstructed with tractography and axonal tracing to reach 19% of reproducibility across methods (Donahue et al., 2016). These comparisons are valuable.

Importantly, when validating dMRI techniques such as diffusion MRI, it is critical to also take into account the limitation of the comparison technique. Histological tracing is more precise than diffusion-weighted imaging tractography because of its spatial resolution and the clear and specific biological mechanisms it obeys. Tracers can be anterograde (i.e., from the neuronal soma to the termination), retrograde (i.e., from the termination to the neuronal soma), monosynaptic (i.e., from the soma to the termination of the same neuron), or polysynaptic (i.e., staining series of neurons). But axonal tracing results are derived from patchy injections in different specimens, and the description of the full connectome is yet impossible. Consequently, the strength of connection will be variable according to anatomical variability across the specimens studied (Croxson et al., 2017) and the absence of information for the areas not or incompletely injected (Schmahmann & Pandya, 2006). Additionally, the strength of the connection derived from axonal tracing remains mostly qualitative (i.e., strong, moderate, or sparse) (Markov et al., 2014) due to the inherent limitation of the method. Hence, discrepancies between ‘gold standard’ and MRI-based approach can be caused by uncertainties in both. As such, the correlations reported could be regarded as encouraging.

These studies demonstrate that however indirect, the comparative approach demonstrated that neuroimaging allows us to record quite reliable signals from humans and non-human primates. In the next section, we explore some of the ways these signals have been used to understand similarities and differences across species.

### 2.2 Identification of qualitative similarities and differences across species

The limitation and validation of the methods apart, approaches using MRI have the ability to assess whether aspects of brain organization obtained in one species also hold in another. Such comparisons can reveal similarities and differences that may shed light on evolutionary processes. Usually, species similarities are interpreted as preserved functions along evolution; whereas more recent phylogenetic changes may explain the differences observed. The quality of the phylogenetic inference stands on the number of species studied as well as the sample size within each species. MRI offers a unique opportunity to access some protected species and collect large samples.

Rilling and colleagues performed some of the pioneering work using MRI for comparative neuroscience to assess standard questions in comparative anatomy. For instance, they used structural MRI scans to compare the relative size of parts of the cortex and of the relative abundance of grey and white matter across different primate species brains (Rilling and Insel, 1998, Rilling and Seligman, 2002). By doing so, they investigated whether the human brain follows the same organisational principles as that of the non-human. Other authors promptly employed the same approach to study the relative size of the human frontal cortex and its proportion of white matter compared to other primates species (Schoenemann et al., 2005, Semendeferi et al., 2002, Sherwood et al., 2005).

The advent of MR-based connectivity measures allowed for the assessment of new evolutionary questions with the cross-species comparison. For instance, the structural organisation of the dorsal frontoparietal connections is comparable in human, chimpanzee, and macaque monkey (Hecht et al., 2015, Thiebaut de Schotten et al., 2011, Thiebaut de Schotten et al., 2012). The functional organisation of the dorsal prefrontal and parietal cortices reveal similar network participation between humans and monkeys (Goulas et al., 2018, Margulies et al., 2009, Mars et al., 2011, Sallet et al., 2013, Vincent et al., 2007). These results indicate that the functions supported by the dorsal frontoparietal network—mostly visuospatial functions involving saccades, spatial working memory, and motor sequences (Parlatini et al., 2017, Petit and Pouget, 2019)—have been preserved along the evolutionary tree.

On the other hand, the most ventral frontoparietal connections differ between the human and chimpanzee (Hecht et al., 2015), suggesting that a change occurred in the functioning of the ventral frontoparietal network since our most recent common ancestor 6 million-years-ago. These differences are of particular interest as the functions supported in the ventral frontoparietal network appear to be more prominent in humans than monkeys (Patel, Sestieri, & Corbetta, 2019). Indeed, meta-analyses of functional MRI in humans indicate that these areas are mostly related to non-spatial function, such as mirror neurons, semantic processing, verbal working memory, phonological processing, decision making, number manipulation, emotion processing, and response inhibition (Parlatini et al., 2017).

Another noticeable difference reported with MRI between human-chimpanzee and macaque is related to frontal-temporal connections. The arcuate fasciculus shows a vastly expanded set of temporal connections in the human compared to the macaque and even the chimpanzee (Rilling et al., 2008), progressively connecting more areas in the frontal and temporal lobes (Eichert et al., 2018, Thiebaut de Schotten et al., 2012). This increase in frontotemporal connectivity volume may be linked to richer symbolic communication existing in humans compared to other primates (Mertz, Surreault, van de Waal, & Botting, 2019), together with changes in the cytoarchitecture of the areas connected (i.e., neuropil volume relative to cell bodies, see Palomero-Gallaghera & Zilles, 2019). In a similar vein, the inferior fronto-occipital fasciculus extends from the frontal cortex to the visual cortex in humans, but some authors argue this tract does not exist in the macaque (Forkel et al., 2014; although some authors have argued this is partly due to a difference in methods employed; Mars and Foxley, 2016, Takemura et al., 2017). Nevertheless, temporal-frontal connections seems to have undergone quite extensive reorganization since the common ancestor of humans and macaques.

Increasing numbers of studies have identified both similarities and differences that were unexpected for researchers. For example, connectivity assessed indirectly using resting-state functional connectivity (Biswal, 2012) revealed that the macaque (Vincent et al., 2007) and the chimpanzee (Rilling et al., 2007) have a default mode network very comparable to the one in humans. These results are comparable to the one reported using tractography and demonstrating a similar crucial hub of connection in core areas of the default mode network (Li et al., 2013). Given the importance of default mode network in humans for thought, autobiographical memory, continuous evaluation and prediction of the environment to guide behaviour, and mentalizing tasks (Catani et al., 2013, Dohmatob et al., in press) these results provide an exciting challenge in understanding the relationship between structure and function across species (Mertz et al., 2019).

Another illustration of the value of the new types of data that can be obtained using imaging is in the debate about the relative size of the prefrontal cortex in the human brain. There has been an active debate about whether the human prefrontal cortex is larger than expected for a primate brain (e.g., Barton and Venditti, 2013a, Barton and Venditti, 2013b; R. E.; Passingham and Smaers, 2014, Semendeferi et al., 2002). This issue is partly due to the poverty of data, with the debate mostly focussed on how the prefrontal cortex was defined in different datasets and the statistical methods employed. Donahue, Glasser, Preuss, Rilling, and Van Essen (2018) recently used MRI data to insert much-needed new data into the debate. By using structural and T1/T2 “myelin” maps of the macaque, chimpanzee, and human brain, they were able to demonstrate that the size of the prefrontal cortex in humans was often underestimated.

Similarly, comparative MRI debunked the myth that brain lateralisation in language areas was unique to humans and revealed that although vervet monkeys, rhesus macaques and bonnet macaques do not show any asymmetries of their planum temporale, chimpanzees do (Gilissen and Hopkins, 2013, Lyn et al., 2011). This unique contribution suggests that planum temporale lateralisation occurred between 6 and 30 million years ago.

The most direct way to investigate whether the brains of two species function in the same way is, of course, to compare task activation of subjects from multiple species doing the same task. Even before comparative neuroimaging, asking different species to perform similar tasks to test their respective cognitive abilities has a long history (see, for instance Joly et al., 2012, Tomasello et al., 1997). One prime example of linking such results to differences in brain organization is provided by Passingham and Wise (2012), who link the evolution of prefrontal cortex to the ability to discover structure in learn of complex tasks (see also Louail, Gilissen, Prat, Garcia, & Bouret, 2019). However, most authors carefully point out that even similar behavioural outcomes in tasks does not mean different species solve the task in the same way. Nevertheless, studies comparing fMRI in humans and macaque monkeys have been successful in comparing visual processing (Vanduffel, Zhu, & Orban, 2014), tool use (Peeters et al., 2009), sequence processing (Wilson et al., 2015), and decision making (Chau et al., 2015), among others. As mentioned in the previous section, functional imaging in non-human primates can also be used fruitfully for longitudinal studies of development or plasticity following lesions (Froudist-Walsh, Lopez-Barroso, Jose Torres-Prioris, Croxson, & Berthier, 2018) and similar studies in marmoset are becoming more frequent (e.g. Hung et al., 2015). However, the difficulty of training non-human primates represent challenges that prevent the exploration of the number of different species required to elaborate solid evolutionary conclusions.

Although exciting, these studies remain quite singular, and comprehensive mapping of similarities and differences across species is still missing. Importantly, most of these studies also rely on rather qualitative assessments of whether brain organization between species is ‘similar’ or ‘different’.

### 2.3 Matching of brains from different species using quantitative measures

The previous section demonstrated some of the potential of comparative MRI to provide much-needed new data and to demonstrate qualitative comparisons of brain organisation across species. However, the real strength of imaging for comparative neuroscience is in its digital nature, which allows for the alignment of features between species producing fields of correspondence.

As a case in point, Van Essen and Dierker (2007) developed an innovative method for anatomical comparison, calculating the deformation field between a macaque and a human brain producing what was called an evolutionary expansion map. This approach was subsequently used by Chaplin, Yu, Soares, Gattass, and Rosa (2013) in a more extensive range of primates, providing a direct comparison of cortical expansion across different primates. Mantini and Corbetta, 2012, Mantini and Hasson, 2012 used the Van Essen expansion in an original way to compare cortical networks defined using resting-state functional MRI, showing one network that seemed to be unique to humans. These results provide quantitative comparisons between species.

Diffusion MRI tractography is one of the most-used technique in comparative MRI, and various authors have sought to assess similarities and differences within a formal statistical framework. For instance, Croxson et al. (2005) manually defined homologous target areas in the frontal lobe of humans and macaques and performed tractography from the bodies of known white matter fibres. For each species, they could statistically compare the distribution of projections of the fibres with the frontal target regions. They then discussed differences in these distributions between species.

The distributions of connections can be directly compared between the species as well. Mars and Foxley, 2016, Mars and Verhagen, 2016 suggested using a non-parametric testing framework to investigate whether the distribution of connections of proposed homologous regions to predefined target regions differed significantly between species. This ‘connectivity fingerprint matching’ approach has been used to compare the whole frontal cortex across the two species (Neubert et al., 2014, Sallet et al., 2013), as well as parts of the temporoparietal cortex (Mars, Sallet, Neubert, & Rushworth, 2013). In a similar vein, Kumar et al. (Kumar, Croxson, & Simonyan, 2016) revealed differences in the connectivity of the laryngeal motor cortex between humans and macaques, showing much higher connectivity with the somatosensory and inferior parietal cortex in humans, possibly related to the role of the laryngeal motor cortex in the production of learned speech in humans.

Connectivity research has also provided fertile ground for an entirely novel approach that highlights the usefulness of having large amounts of similar data available from a range of species (see an illustration in Fig. 1). Sporns and colleagues have investigated how the organisation of brain connectivity might satisfy various constraints that work on biological systems. For instance, the expensive nature of brain tissue means that optimal wiring of the brain is a compromise between creating many connections to produce the shortest possible "routes" between brain areas and pruning connections to keep the energetic demands of the brain feasible (Bullmore and Sporns, 2012, Karolis et al., 2018). Statistical analysis of the whole-brain connection maps—termed connectomes—of various species demonstrates that these principles hold across vastly different brains (van den Heuvel, Bullmore, & Sporns, 2016). Connectome approaches provide some of the most prominent examples of using large-scale statistical analyses to study the principles of brain organisation across species (e.g., Li et al., 2013).

Overall, neuroimaging allows one to collect data from enough samples and to analyse these data using powerful statistical frameworks. We are beginning to see the potential of these approaches.

## 3 Part III: what do we need to take the next steps?

In this third section, we discuss some of the steps that we believe will help push comparative neuroimaging towards the goal of large-scale comparisons.

### 3.1 Combine resources

Large-scale comparative neuroimaging of many species is an enormous challenge, especially for a field still in its infancy. Moreover, many of the species that are of interest to study are rare, and samples are challenging to obtain. Therefore, it is essential that researchers share datasets.

As discussed above, the nature of MRI data makes it very suitable for sharing. In human neuroscience, some very large-scale projects, such as the Human Connectome Project (Van Essen et al., 2013, Van Essen and Ugurbil, 2012), the 1000 Functional Connectomes (Biswal et al., 2010), and the UK Biobank (Collins, 2012, Palmer, 2007) have set new standards for data sharing, including novel online infrastructures and new quality control procedures (Boubela et al., 2015, Burgess et al., 2016, Herrick et al., 2014, Marcus et al., 2013, Szalkai et al., 2017). Comparative neuroimaging currently has no such resources, but many recent initiatives are encouraging (see Table 1). Building on the infrastructure of the 1000 Functional Connectomes Project, Milham and colleagues recently launched the Primate Data Exchange (PRIME-DE) initiative, which hosts in-vivo MRI data from macaques collected by more than 20 different labs (Milham et al., 2018). Roberto Toro's Brain Catalogue hosts structural data from post-mortem samples of primates and other animals (Heuer et al., 2019). Finally, the popularity of diffusion MRI and its successful application to post-mortem data allows various groups to share these data from a range of primates, with the National Chimpanzee Brain Resource (https://www.chimpanzeebrain.org) as a prime example.

**Table 1:** Non-human primate MRI data resources.

Resource	Genus/species	Main modalities	Web location	Corresponding publication(s)
Brain Ark	Dolphin, Tasmanian devil, Thylacine, primates expected	Diffusion MRI	brainark.org	Berns et al. (2015) Berns and Ashwell (2017)
Brain Catalogue	Various, including primates and other mammals	T1 scans	braincatalogue.org	
BALSA	Macaque, chimpanzee	Statistical maps, atlases, results files	balsa.wustl.edu	Van Essen et al. (2017)
Duke University Center for In Vivo Microscopy	Macaque and rodents	Various	civm.duhs.duke.edu/SharedData/DataSupplements.htm	
JMC Primates Brain Imaging Repository	Various primates	T2 scans, diffusion MRI	j-monkey.jp/BIR/index_e.html	Sakai et al. (2018)
MaMi collection (Assaf & Yovel)	>100 species, including primates	Diffusion MRI		
National Chimpanzee Brain Resource	Chimpanzee	T1 scans, T2 scans, diffusion MRI	chimpanzeebrain.org	Various
Neuroecology lab collection	Various primates	Diffusion MRI, T1 scans, results files	neuroecologylab.org	
Neurovault	Macaque	Unthresholded statistical maps	neurovault.org	
PRIME-DE	Macaque	Resting state fMRI	http://fcon_1000.projects.nitrc.org/indi/indiPRIME.html	Milham et al. (2018)
UNC-Wisconsin Neurodevelopment rhesus MRI database	Macaque	T1 scans, T2 scans, diffusion MRI	nitrc.org/projects/uncuw_macevmri	Young et al. (2017)

These initiatives show the willingness of researchers to share comparative data. However, they also demonstrate the inevitable downsides of collecting data from different laboratories. The data available are collected from samples from different sources, and the animals are anaesthetised or the samples are fixed and preserved in different ways. The data are obtained from different scanners and using different, often non-standard, protocols. The effects of these differences on the results are not always known, although some studies are starting to investigate these issues systematically (e.g., Xu et al., 2018). Combining data from in-vivo and ex-vivo scans presents challenges due to the changes in samples shape and tissue properties related to post-mortem fixations, while different ex-vivo scans themselves might differ in their fixation delays and protocols, all which might influence the signal obtained from the samples (Dawe et al., 2009, Widjaja et al., 2009). Aggravating this issue is that the samples that are compared in a comparative study differ in volume and weight—even within the primate order brain weight varies from the less-than-100 g mouse lemur brain to the 1.3 kg human brain—and therefore are by necessity scanned at resolutions that differ either in absolute terms or in relation to the brain. Some of these problems can be addressed by adjusting analysis parameters, for instance, some researchers change tractography settings for comparative diffusion MRI studies, but the full effects of such adjustments have not been explored systematically.

Sharing of comparative data is associated with organisational challenges other than those associated with sharing human data. Data obtained from animals can be more controversial than human data. These data are acquired under different legal and regulatory restrictions that affect data sharing, and the data are costlier and harder to obtain. Because the field is young, many of the labs sharing these types of data are headed by young PIs that might be reluctant to share without the possibility of credit. Whichever position one takes in these debates, it is essential for these issues to be acknowledged. For these reasons, rather than an “everybody shares all data without limitations”-approach, which might not be feasible, a more tailored approach in which the difficulties and benefits for all involved are considered is commendable. This limitation in no way diminishes commitment to open science; it simply acknowledges an issue to come. The PRIME-DE initiative provides an excellent proof of concept on dealing this limitation, as different datasets are accessible under a variety of different licences (Milham et al., 2018).

Calls to action: Share data into large-scale initiatives that allow flexibility to accommodate the constraints of any particular data set; understand how differences in acquisition parameters across sites affect our data, and to what extent datasets can be combined.

### 3.2 Develop and share tools specialised for comparative MRI

Some standardisation of analysis protocols and a better understanding of the analyses' biases would facilitate the exchange of data and the interpretation of results across the different groups. Such a consensus would also help researchers to learn which differences between methods and data quality are crucial and which are not (for instance, how one process resting state data is increasingly thought to be of importance, Bright, Tench, & Murphy, 2017). However, one should not spend much time having a sterile discussion on which step is best for any dataset if it does not affect the results much.

Human neuroimaging has benefitted from standardised preprocessing and reporting pipelines. For instance, the Human Connectome Project released data in a ‘minimally preprocessed’ format (Glasser et al., 2013) that has resulted in a host of connectivity papers using the same pipeline and results that are more comparable across studies. FSL's FEAT tool produces a standardised report that allows authors to describe their preprocessing using comparable terminology. Comparable standardised pipelines are not available for non-human primate imaging. Moreover, since most tools for neuroimaging are tailored to the human brain, they often require quite substantial adaptation to be suitable for non-human primate data. Hence, there is currently some inconsistency in the analysis strategies, even across studies of the same species (such as the macaque monkey), that may lead to contrasting results, disputes, and hamper progress in science.

A preliminary attempt of an inventory of the tools to analyse imaging data from non-human primates is listed in Table 2 but is also by no means exhaustive. A version will be regularly updated online (www.neuroecologylab.org). Software packages dedicated to human brain research such as SPM and FSL have often been adapted on a case by case basis. There are also recipes for adapting Freesurfer's RECON pipeline for non-human primate data online, as well as adjustments of the HCP's minimal preprocessing pipeline. Since various registration algorithms rely on priors regarding the size and shape of the brain, they often need modifications; the Advanced Normalization Tools (ANTs) by Avants and colleagues (Avants, Epstein, Grossman, & Gee, 2008) are often used in this context.

**Table 2:** Some analysis packages and tools used in the analysis of comparative MRI data-human primate MRI.

Resource	Description	Web location	Corresponding publication(s)
AFNI	fMRI analysis package, primarily focused on the human	afni.nimh.nih.gov	Cox et al. (1996)
ANTs	Normalization tools for MRI data	stnava.github.io/ANTs	Avants et al. (2014)
CARET/Connectome Workbench	Visualization and discovery of data for Human Connectome Project	https://www.humanconnectome.org/software/connectome-workbench	
CIVET	MRI image processing package, primarily focused on the human	mcin-cnim.ca/technology/civet	
CONN Toolbox	Functional connectivity toolbox for Matlab	sites.google.com/view/conn	Whitfield-Gabrieli and Nieto-Castanon (2012)
Freesurfer	MRI image processing focusing on cortical surface analysis, primarily focused on the human but with adaptations for non-human primate online	surfer.nmr.mgh.harvard.edu	Fischl, 2012, Fischl et al., 1999 Dale, Fischl, and Sereno (1999)
FSL	MRI image processing package, primarily focused on the human	fmrib.ox.ac.uk/fsl	Smith et al. (2004)
HCP preprocessing pipelines	Pipelines for processing MRI data, including freesurfer-based reconstructions, primarily focused on the human	humanconnectome.org	Glasser et al. (2013)
Minc-toolkit	Packages for manipulating imaging data in minc format	Github.com/BIC-MNI/minc-toolkit-v2	
MR Comparative Analysis Toolbox (Mr Cat)	Collection of wrappers (mostly around FSL) for preprocessing of non-human data and unique scripts for comparative analyses	neuroecologylab.org	
SPM	MRI image processing package, focused on the human	www.fil.ion.ucl.ac.uk/spm	

The sharing of the analysis code, for instance on GitHub, is becoming increasingly common and should be encouraged. The knock-on effect of developing more of these tools, and of a second imperative—making them easier to use, so one does not have to be an expert in the field to use them—is that we will open up a wealth of possibilities and collaboration. People who do not consider themselves “experts” in the field of neuroimaging will be more and more willing to contribute to comparative neuroimaging, and, in turn, will help answer new questions.

Another way to facilitate communication of results across groups is to adopt a common template space in which results will be reported. Human neuroimaging has benefitted hugely from the adaption of MNI standard space, which has facilitated databases for meta-analyses such as NeuroSynth and BrainMap (Fox & Lancaster, 2002). For the most commonly used non-human primate, the macaque, a number of different templates have been suggested (Table 3). The recently proposed NMT atlas (Seidlitz et al., 2018) anticipates the many formats and MRI-related data types, featuring an anatomical template, surface representations, and registered atlases (Reveley et al., 2017). Templates for other species are as yet more scarce, although some are available, such as the Riken's BSI-NI atlas for the marmoset and various templates for rodents (e.g., Hikishima et al., 2017, Valdes-Hernandez et al., 2011). To facilitate comparison across species, standardised techniques for template creation are essential, with most groups employing ANTs.

**Table 3:** Some templates and atlases for non-human primate MRI.

Resource	Species	Description	Web location	Corresponding publication(s)
Brain/MINDS 3D digital marmoset brain atlas	Marmoset	Atlas and coregistered histology	https://www.brainminds.riken.jp/reference-atlas-data	Woodward et al. (2018)
BSI-NI marmoset	Marmoset	Template and atlas	Brainatlas.brain.riken.jp/marmoset/modules/xoonips/listitem.php?index_id=3	Hikishima et al. (2011)
D99	Macaque	Template and atlas	Afni.nimh.nih.gov/Macaque	Reveley et al. (2017)
F99	Macaque	Template and atlas	brainvis.wustl.edu/wiki/index.php/Caret:Atlases	Van Essen et al., 2012
McLaren template	Macaque	Template coregistered to Saleem atlas	Brainmap.wisc.edu/monkey.html	McLaren et al. (2009)
MIRCen Mouse Lemur Atlas	Mouse lemur	MRI template and atlas	https://www.nitrc.org/projects/mouselemuratlas	
MNI monkey space	Macaque	Template with coregistered Paxinos atlas	Bic.mni.mcgill.ca/ServicesAtlases/Macaque	Frey et al. (2011)
NIH Marmoset Brain Atlas	Marmoset	Template and atlas	https://github.com/NIHMarmosetBrainAtlas/NIH_Marmoset_Atlas	Liu et al. (2018)
NIMH Macaque Template	Macaque	Template, including surfaces	Github.com/jms290/NMT	Seidlitz et al. (2018)
VALiDATe29 squirrel monkey brain atlas	Squirrel monkey	Various modalities including T1 and diffusion MRI	https://www.nitrc.org/projects/validate29/	Schilling et al. (2017)
Yerkes19 and Yerkes29	Macaque and chimpanzee	Templates		

Calls to action: Talk to each other to use similar acquisition and processing methods where possible – reach a consensus as a field. Share pipelines and analysis tools in a central resource. Find a way to make the field more user-friendly.

### 3.3 Agree on a common framework

With new data and new types of analyses comes another, perhaps more unappreciated, challenge: the need for the field to agree on a common framework. Researchers will have to agree on questions to study and what criteria will be used to judge whether answers provided by a given study are considered satisfactory.

As pointed out in the position paper by Striedter et al. (2014), one crucial step is to create maps of brain organisation for various species. Ideally, we should start with maps such as those of mammalian early sensory areas presented by Krubitzer to argue for a universal plan of mammalian brain organisation (Krubitzer, 2007). MRI will allow us to create such maps but—crucially—will also allow us to compare these maps quantitatively.

When comparing maps of different brains, the apparent problem is that they differ drastically in size and shape, even if some of the features of interest remain constant. For instance, to understand whether the connections between homologous brain regions have changed, it helps to be able to overlay homologous brain regions of the species of interest and then compare the connectivity matrices. Such a ‘common space approach’ allows us to study features of interest across brains while holding all other irrelevant features constant (Mars, Eichert, Jbabdi, Verhagen, & Rushworth, 2018).

In effect, this approach was taken by Van Essen and colleagues when comparing relative expansion (the feature of interest) by overlaying brains cased on homologous sulcal anatomy (the irrelevant feature). Other studies used the profile of connections between areas to compare brain organisation between species (cf. Mars, Verhagen, et al., 2016). For instance, when comparing the organisation of the frontal cortex, Sallet et al. (2013) determined the profile of connections in human areas, showing that each area had a unique set of connections. They then compared the connections of areas in the frontal cortex of the macaque with areas thought to be homologous to the human areas. By comparing the similarity in connectivity profiles across species, each human dorsal frontal region could be matched to a region in the macaque. Thus, this approach brought both brains into a single connectivity space defined by the homologous target areas (see also Mars et al., 2013, Neubert et al., 2014, Neubert et al., 2015).

This approach can be generalised. Recently, Mars et al. (2018) described the organisation of each part of the human and macaque cortex in terms of its connectivity with 39 white matter tracts as identified using dMRI tractography. Each of these tracts can be defined based on the anatomical location of their tract bodies, even if the projections of the tracts to the cortex differ across species. They called the matrices of each species’ connectivity between each part of the cortical surface vertices and all white matter tracts the connectivity blueprints of two brains. Since the tract dimensions of these two blueprints are identical, it is, in effect, a common space in which both cortices can be described. For each part of the macaque cortex, it was then possible to identify the part of the human cortex that had the most similar connections. Taking the approach even further, they mapped the degree to which the human brain matched the macaque brain, creating a probabilistic map of variations between the two brains.

The type of analysis allowed by the connectivity blueprint analysis is only achievable with the advances in digital data analysis. Importantly, they also allow us to ask different questions of our comparative data. Rather than searching for homologs across brains, it provides a continuous dimension of similarity and differences between brains. For instance, if one aligns two brains using sulcal anatomy as done by Van Essen and colleagues, and using connectivity blueprints as done by Mars and colleagues, it is of interest to see to what extent the results overlap. When two methods do not converge, this would suggest a reorganisation between the two lineages. As an early example of this approach, Eichert et al. (2018) demonstrate that the Van Essen cortical expansion cannot account for differences in the projections of the arcuate fascicle between the human and the macaque, arguing for an expansion of connections into new cortical territories in one brain compared to the other.^2^

We believe that in order to large-scale comparative neuroscience to reach its full potential, it is essential that the field agrees on a framework of understanding. The ‘common space approach’ advocated above is one option that fully exploits the possibilities offered by using neuroimaging technique. However, we of course do not mean to imply that comparative neuroscience should exist separated from other evolutionary and comparative work—indeed progress can only made if different subfields, using different techniques, learn to communicate with one another and learn from each other's results.

Calls to action: Agree on a framework of questions and answers that allow us to fully exploit the opportunities offered by comparative MRI, while incorporating and respecting the frameworks and results obtained in current comparative neuroscience.

## 4 Conclusions

We have argued that neuroimaging provides unique opportunities for comparative neuroscience to move into large-scale studies that are essential to study principles of species diversity. Neuroimaging should not be considered as a replacement for established anatomical techniques, but rather as a new promising tool with limitations. Opportunities lie in the ease with which whole-brain, multi-modal data can repeatedly be obtained in a non-invasive and non-destructive manner. The whole-brain and digital nature of the data allow a new way of aligning brains within a common space, providing a formal framework for the comparison of cortical maps. We cannot succeed without the community's willingness to share data and analysis protocols and to agree on a joint approach for moving forward. Sharing and working together will make the future of comparative neuroimaging very bright indeed.

## Footnotes

2 Interestingly, this approach can also be used to investigate whether some aspects of anatomical organization as measured by MRI might correlate with other anatomical features that could present possible confounds, such as increasing gyral bias of tractography results in more encephalized species (cf. Reveley et al., 2015).
